# Tracking and analyzing the spatio-temporal changes of rice planting structure in Poyang Lake using multi-model fusion method with sentinel-2 multi temporal data

**DOI:** 10.1371/journal.pone.0320781

**Published:** 2025-04-07

**Authors:** Fenglan Pi, Yang Chen, Guoqing Huang, Shaohua Lei, Dalin Hong, Ning Ding, Yuanzhi Shi

**Affiliations:** The National Key Laboratory of Water Disaster Prevention, Nanjing Hydraulic Research Institute, Nanjing, China; Sathyabama Institute of Science and Technology (Deemed to be University), INDIA

## Abstract

Accurate and efficient extraction of rice planting structures, coupled with comprehensive analysis of their spatiotemporal dynamics and driving factors, is crucial for rice yield estimation and optimized water resource management in the Poyang Lake region. However, traditional approaches face significant limitations: single machine learning models often yield insufficient classification accuracy, while existing fusion models typically involve complex processing workflows and exhibit low computational efficiency. To address these challenges, this study developed an efficient and simplified fusion model based on a scoring strategy to determine rice planting structures from 2018 to 2023, followed by an in-depth analysis of their spatiotemporal patterns and underlying drivers. The evaluation results demonstrated that four individual classification models of K-Nearest Neighbors (KNN), Random Forest (RF), Support Vector Machine (SVM), and Gradient Boosting Decision Tree (GBDT) achieved Overall Accuracy of 85.29%–90.07%, Kappa coefficients of 0.786–0.855, User Accuracy of 80.51%–93.02%, and Mapping Accuracy of 80.87%–92.63%. The proposed scoring-based fusion model significantly enhanced these metrics, improving Overall Accuracy by 3.36%–9.16%, Kappa coefficient by 5.15%–14.38%, User Accuracy by 0.37%–11.13%, and Mapping Accuracy by 0.48%–10.71%. Spatiotemporal analysis revealed distinct trends in rice cultivation patterns: single-cropping rice and regenerated rice showed consistent expansion, both in planting area and proportion, with a spatial tendency towards flat regions. Conversely, double-cropping rice exhibited a gradual decline, with its cultivation areas contracting towards the central lake region. These shifts were primarily driven by socioeconomic factors, particularly rural labor migration and rising fertilizer prices, which have incentivized farmers to adopt less labor-intensive and lower-input cultivation systems, such as single-cropping and regenerated rice. The findings offer a novel methodological framework for precise extraction of crop planting structures, and a scientific foundation for local governments to develop targeted water resource management strategies.

## 1. Introduction

The escalating demand for food in China has become increasingly pronounced, driven by the nation’s rapid population expansion and accelerated urbanization processes. Rice (Oryza sativa L.), serving as a staple cereal crop in China, covers approximately 160 million hectares of cultivated land, representing 27.4% of the global rice cultivation area. Therefore, maintaining and stabilizing rice cultivation areas has become a critical strategy for ensuring national food security [1]. The Poyang Lake region, blessed with abundant rainfall and fertile soil conducive to rice cultivation, has emerged as a pivotal rice-producing hub in Jiangxi Province. This region has three distinct rice planting patterns, encompassing single-cropping rice, double-cropping rice, and regenerated rice, which increases the difficulty of extracting different types of rice area [2]. In recent years, factors such as regional climate change, shifts in agricultural policies, changes in rural labor force, and the widespread impacts of the COVID-19 pandemic have profoundly affected the types and extent/acreage of rice cultivation in the region [3,4]. Accurately extracting the cultivated area of different types of rice and analyzing the change trends and influencing factors, is crucial for the government to effectively allocate water resources and develop agricultural policies that ensure food security in the Poyang Lake region. The traditional method for extracting rice planting structures predominantly relies on manual field surveys, which presents significant limitations in terms of temporal efficiency, labor requirements, and spatial coverage. Consequently, the integration of high spatiotemporal resolution satellite remote sensing imagery with advanced image processing algorithms has emerged as a transformative approach for precise extraction of rice cultivation areas across diverse geographical regions. This methodology offers distinct advantages over traditional field surveys, including extensive spatial coverage, frequent temporal observation capabilities, and cost-effective operational characteristics [5–7].

In recent years, with the gradual development of geo-remote sensing information and artificial intelligence technologies, various data products including the Moderate Resolution Imaging Spectroradiometer (MODIS), Landsat, and Sentinel [4,8,9], and different methods including the single imagery method, time-series imageries method, and imageries combined with census data method were widely used for extracting crop planting structures [10]. However, these products and methods each exhibit distinct advantages and disadvantages, which constrain their capacity to fully address customer requirements. For example, while Landsat and Sentinel satellite imagery offer high spatial resolution, their limited temporal resolution and occasional data quality issues often lead to the unavailability of images during critical crop growth stages, which consequently compromising the accuracy of crop planting structure mapping [11]. The imagery from MODIS, characterized by its high temporal resolution and extensive coverage, is particularly well-suited for large-scale regional mapping. Nonetheless, its relatively low spatial resolution poses challenges in delivering detailed vegetation classification in regions with complex terrain [12]. Therefore, previous studies have frequently relied on integrating multiple data sources and combining tailored research methodologies to accurately extract crop planting structures across diverse conditions. Tian et al (2018) demonstrated that combining Sentinel-1A and Landsat-8 data for mapping multi-season paddy rice in the Poyang Lake Plain could achieve higher accuracy compared to using single satellite data alone [4]. Chen et al (2023) effectively integrated MODIS image data with differential algorithms, spectral mutation algorithms, and threshold methods to extract the planting areas of major crops in the Yellow River Basin, and achieved robust and reliable results [13]. In addition, Tang et al (2022) mapped typical cultivated areas in Inner Mongolia based on single-phase Sentinel-2 images fused by the pixel samples and the neighborhood information [14].

Machine learning algorithms, such as decision trees (DT), support vector machines (SVM), random forests (RF), and artificial neural networks (ANNs), are capable of efficiently and accurately processing vast amounts of remote sensing image data from diverse sources and resolutions, making them widely adopted in land cover mapping applications [15–17]. For instance, Zhang et al (2020) evaluated the accuracy of eight machine learning models for rice paddy mapping and identified the two-dimensional convolutional neural network as the most accurate, subsequently utilizing it to extract planting areas in Chongqing, Southwestern China [18]. Kabolizadeh et al (2023) employed multi-temporal images and machine learning algorithms to accurately extract the planting areas of main crops in the Khuzestan plain, Iran [19]. Zhong et al. (2019) demonstrated that the Mapping Accuracy of crop planting structures in Yolo County, California, reached 85.54% when employing one-dimensional convolutional (Conv1D) layers, surpassing the 84.17% accuracy achieved using gradient boosting machines [20]. Ensemble learning, an advanced strategy in machine learning, offers numerous advantages over traditional single-model approaches, including enhanced prediction accuracy, superior generalization performance, and faster training speeds [21,22]. Li et al. (2020) utilized ensemble learning to map wetland types in the coastal landscape of the Manning River Estuary, Australia, achieving significantly higher accuracy compared to using a single machine learning algorithm [23]. Furthermore, Yu et al (2024) proposed a simple multimodal fusion method to enhance winter wheat classification accuracy based on three conventional machine learning algorithms [37].

To our knowledge, rice cultivation in Poyang Lake region is predominantly conducted by small-scale peasant farmers, characterized by a high degree of randomness and complexity in cultivation practices across relatively small field areas. Consequently, higher spatial resolution and long-sequence satellite images, along with advanced classification methods are required to accurately extract its planting structure. Moreover, the temporal and spatial changes in rice cultivation structures in the Poyang Lake region over the past six years, driven by factors such as climate and policy changes, as well as population mobility, have not yet been thoroughly investigated. Therefore, the main objectives of this study were to: (1) compare the accuracy of several commonly used machine learning models and their fusion model based on Sentinel-2 image data in extracting rice planting structure; (2) map the distribution of different rice varieties from 2018 to 2023 using the highest-accuracy model; (3) analyze the key factors driving the spatio-temporal variations in rice planting structure in the Poyang Lake region.

## 2. Materials and methods

### 2.1. Study area

**Fig 1:**
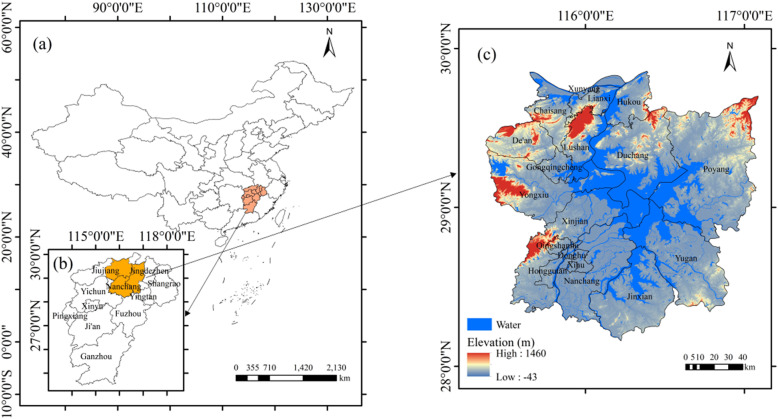
Geographical location and spatial distribution of the study area.

The Poyang Lake region (28°11’ − 29°51’N, 115°31’ − 117°06’E) is located in Jiangxi Province, China (Fig 1a). The region belongs to subtropical warm and humid monsoon climate, with mean annual temperatures ranging from 16.5 °C to 17.8°C and an average annual precipitation of 1570 mm. The Poyang Lake area encompasses 19 counties and districts, covering a total area of 21,095 km^2^, which accounts for approximately 12.63% of Jiangxi Province’s total land area (Fig 1b). The topography surrounding the lake and in the southeastern region is predominantly flat, with abundant water resources and favorable conditions for rice cultivation. The agricultural system in this region primarily consists of double-cropping rice, single-cropping rice (including mid-season rice and single-cropping late rice), and regenerated rice. Double-cropping early rice is typically transplanted from mid-to-late April to early May and reaches maturity in early to mid-July. Double-cropping late rice is transplanted from late July to early August and matures from mid-to-late October to early November. Medium-season rice is transplanted from mid-to-late May to early June and matures from early to late September. Single-cropping late rice is transplanted in early to mid-July and matures in mid-to-late October. Regenerated rice is transplanted in late April to early May, with the first crop maturing and being harvested in August, and the regenerated crop maturing from late October to early November. The intensive rice cultivation system has established the area as one of the most significant rice production bases in Jiangxi province and even in China.

### 2.2. Data collection

#### 2.2.1. Remote sensing data.

The satellite image data used in this study derived from the Sentinel-2 satellites (Sentinel-2A, B) operated by the European Space Agency (ESA). These twin satellites operate in a sun-synchronous polar orbit with a 180° orbital phasing difference, providing a combined temporal resolution of 5 days for image acquisition. The Multi-Spectral Instrument (MSI) onboard the Sentinel-2 satellites operates at an altitude of 786 km, featuring a swath width of 290 km and 13 spectral bands spanning the visible (VIS), near-infrared (NIR), and shortwave infrared (SWIR) regions. The spatial resolution varies across different spectral bands, with three distinct levels: 10 m, 20 m, and 60 m, as detailed in Table 1. Therefore, Sentinel-2 satellite data exhibits significant advantages in the extraction of crop planting structure due to the high spatial and temporal resolution, multi-spectral imaging capabilities, open-data policy, and data consistency.

**Table 1:** The bands attributes of Sentinel-2 data.

Bands	Center wavelength (nm)	Spatial resolution (m)
Band1-Coastalaerosol	443	60
Band2-Blue	490	10
Band3-Green	560	10
Band4-Red	665	10
Band5-Vegetationrededge	705	20
Band6-Vegetationrededge	740	20
Band7-Vegetationrededge	783	20
Band8-NIR	842	10
Band8a-Narrow NIR	865	60
Band9-Watervapour	945	20
Band10-SWIR-Cirrus	1380	60
Band11-SWIR	1610	20
Band12-SWIR	2190	20

In this study, satellite image preprocessing was implemented on the Google Earth Engine (GEE) cloud computing platform, which provides advanced geospatial processing capabilities. The preprocessing workflow incorporated three essential corrections: (1) orthorectification to remove geometric distortions, (2) geometric correction for spatial alignment, and (3) atmospheric correction to eliminate atmospheric interference effects. Following these corrections, band arithmetic operations were performed to derive the Normalized Difference Vegetation Index (NDVI) through the following standardized formula:


$$NDVI=\frac{NIR-RED}{NIR+RED}$$ (1)

where NIR is the Near-Infrared spectral band of Band 8, and RED is the Red spectral band of Band 4. The maximum Normalized Difference Vegetation Index (NDVI) values for each pixel across multiple images within a month are extracted using ArcGIS software. This process is repeated to generate monthly NDVI data for each pixel throughout the year, spanning 12 months. Eventually, this approach leads to the construction of an NDVI time-series dataset with a spatial resolution of 10 m, covering the period from 2018 to 2023.

#### 2.2.2. Training and validation samples.

**Fig 2:**
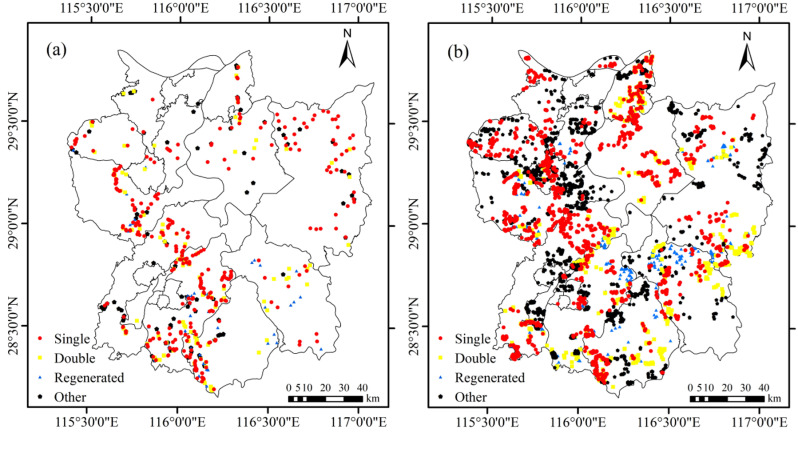
The labeling data of field survey (a) and visual interpretation (b) in the study region. Single: single-cropping rice; Double: double-cropping rice; Regenerated: regenerated rice; Other: other land cover types.

**Fig 3:**
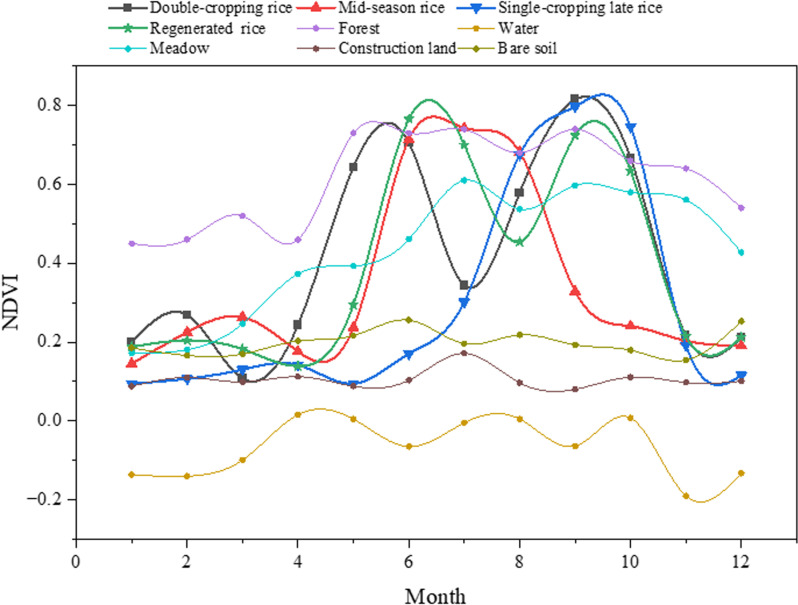
The time series curves of normalized difference vegetation index (NDVI) for different types of rice and other land cover types.

The ground reference data collected in the Poyang Lake region was mainly used for both machine learning model development and accuracy validation. These labeled samples were mainly obtained through field surveys and visual interpretation of high-resolution satellite imagery. Based on extensive field investigations, we established a comprehensive land cover classification system consisting of five primary categories: (1) double-cropping rice, (2) single-cropping rice (including both mid-season and single-cropping late rice), (3) regenerated rice, (4) other vegetation types, and (5) non-vegetated surfaces (comprising water bodies, built-up areas, and bare land). The field survey was conducted in August 2022, coinciding with the phenological stage when different rice cultivation types exhibit maximal spectral differentiation. Through systematic ground truthing, we collected a comprehensive set of reference samples, including 316 single-cropping rice points, 179 double-cropping rice points, 115 regenerated rice points, and 162 non-rice land cover points (Fig 2a). Subsequently, additional reference samples were acquired through visual interpretation of high-resolution imagery available on Google Earth Pro platform. This supplementary dataset comprised 1794 single-cropping rice samples, 878 double-cropping rice samples, 407 regenerated rice samples, and 1553 non-rice land cover samples (Fig 2b). Ultimately, a total of 5404 sample points were obtained in 2022, including 2110 single-cropping rice samples, 1057 double-cropping rice samples, 522 regenerated rice samples, and 1715 other land cover types. These samples were randomly divided into training and validation sets at a ratio of 7:3. The NDVI time series curves of several land types were obtained from field survey sample points and corresponding satellite images (Fig 3).

#### 2.2.3. Statistical data collection.

In this study, the average slope of each administrative region was utilized as an indicator of terrain flatness. High-resolution Digital Elevation Model (DEM) data were acquired from the Advanced Land Observing Satellite (ALOS) and processed using ArcGIS software to calculate the average slope for each administrative region. In addition, the elevation of individual rice fields was extracted using ArcGIS and DEM data. Climate data, including average temperature and precipitation for the Poyang Lake region, were derived from meteorological station records in Jiujiang, Nanchang, and Shangrao cities, accessed through the public database of the Jiangxi Provincial Meteorological Bureau (http://jx.cma.gov.cn/). Socio-economic data, such as the monthly average income of migrant workers and the total number of migrant workers nationwide, were obtained from the “Migrant Worker Monitoring and Survey Report from 2018–2023” published by the National Bureau of Statistics (https://www.stats.gov.cn/). Furthermore, the China fertilizer wholesale price composite index (CFCI) and the minimum purchase price of rice were sourced from the National Development and Reform Commission of the People’s Republic of China (https://www.ndrc.gov.cn/). Data on the China agrochemical price index were retrieved from China National Pesticide Industry Association (https://www.ccpia.org.cn/).

### 2.3. Mapping rice planting structure

**Fig 4:**
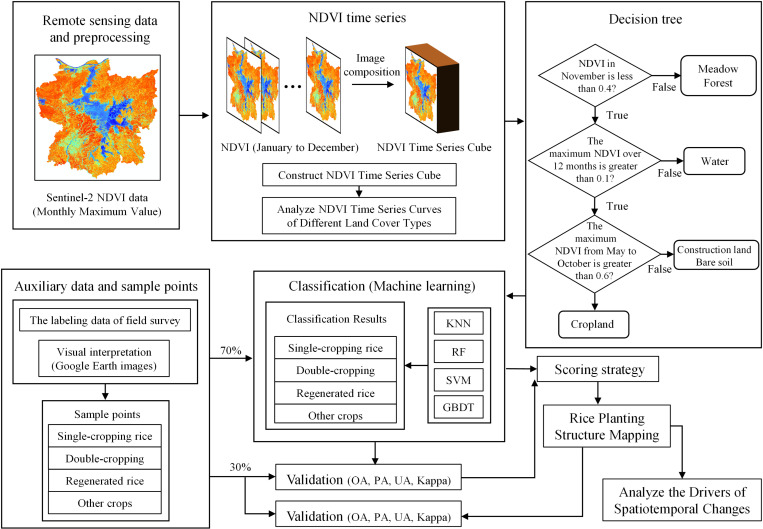
The technology roadmap of this study. KNN: K-Nearest Neighbor; RF: Random Forest; SVM: Support Vector Machine.

The overall workflow of this study is shown in Fig 4, comprising four main steps: (1) The decision tree method was employed to eliminate non-cultivated land types based on the characteristics of NDVI time-series curves for different land cover types; (2) The rice species and validation accuracy of four individual machine learning models for extracting rice planting structure were obtained; (3) A classification method was developed based on a scoring strategy from the validation accuracy of individual machine learning models; (4) The rice planting structure from 2018–2023 was extracted, and the driving factors of its spatio-temporal changes were analyzed.

#### 2.3.1. Decision tree.

As illustrated in Fig 3, the temporal NDVI variation characteristics of different sample types are clearly distinguishable. The NDVI time series curves for double-season rice and regenerated rice exhibit clear double-peak patterns. Specifically, double-season rice exhibits peaks in June and September, with a trough in July, while regenerated rice shows peaks in July and September, with a trough in August. Mid-season rice and single-cropping late rice show distinct single-peak patterns, with NDVI values declining rapidly after reaching their peaks. By November, rice is fully harvested, resulting in a significant drop in NDVI values to below 0.3. In contrast, meadow and forests maintain relatively high NDVI values during this period, exceeding 0.5. Water bodies consistently have NDVI values below 0.1 throughout the year, making them easily distinguishable from farmland. Construction land and bare soil exhibit consistently low NDVI values below 0.25 year-round, with minimal fluctuation. To streamline the dataset for machine learning and optimize workspace efficiency, a decision tree classification rule was first constructed to exclude meadows, forests, water bodies, construction land, and bare soil (Fig 4). Specifically, lands with an NDVI value greater than 0.4 in November were classified as grassland and forest, lands with a maximum NDVI value below 0.1 throughout the year were classified as water bodies, and lands with a maximum NDVI value below 0.6 from May to October were classified as construction land and bare soil.

#### 2.3.2. Machine learning model.

K-Nearest Neighbor (KNN) is a robust classification method widely used in data mining and pattern recognition. The core principle of KNN is that if a majority of the K most similar samples in the feature space belong to a specific category, the given sample is also assigned to that category. In other words, each sample is represented by its K nearest neighbors [24,25]. Therefore, during classification, KNN determines the category of a sample solely based on the categories of its nearest neighbors [25]. In this study, the grid search method integrated in scikit-learn library in Python was employed to optimize the parameters of the K-Nearest Neighbors (KNN) algorithm. The n_neighbors parameter was set to 7, the weight to ‘uniform’, the metric to ‘minkowski’, the p to 2, the algorithm to ‘auto’, the leaf size to 50, and n_jobs to ‘None’.

Random Forest (RF) is an advanced ensemble learning algorithm that builds upon the foundation of decision trees, significantly enhancing prediction accuracy and stability through the aggregation of multiple decision tree predictions [26]. The algorithm’s inherent randomness manifests in two key aspects: random sample selection and random feature selection. The former employs a bootstrapping technique to construct diverse sub-decision trees, while the latter involves randomly selecting feature subsets as candidate sets for each sub-decision tree construction. These dual randomization mechanisms effectively mitigate overfitting risks, endowing random forests with superior robustness and noise tolerance compared to conventional decision tree approaches. Furthermore, this methodology demonstrates exceptional capability in handling high-dimensional feature spaces, making it particularly suitable for complex datasets with numerous variables [27]. In this study, the grid search method integrated in scikit-learn library in Python was employed to optimize the parameters of the Random Forest (RF) algorithm. The n_estimators parameter was set to 500, the max_depth to 5, the max_features to ‘None’, and the random_state to 1 [28].

Support Vector Machine (SVM) is a supervised learning model rooted in statistical learning theory [29]. Known for its exceptional robustness, SVM is particularly advantageous in solving nonlinear classification problems, processing datasets with limited training samples, and handling high-dimensional feature spaces [30]. The algorithm employs kernel functions to map sample data from the original feature space into a higher-dimensional space, where it constructs an optimal separating hyperplane. This hyperplane maximizes the margin between different classes while minimizing classification errors, thereby achieving effective discrimination among sample categories [31]. In this study, the grid search method integrated in scikit-learn library in Python was employed to optimize the parameters of the Support Vector Machine (SVM) algorithm. The kernel parameter was set to ‘rbf’, the gamma to 0.8, the decision_function_shape to ‘ovr’, and the C to 0.95 [32].

The Gradient Boosting Decision Tree (GBDT) classifier is an ensemble machine learning algorithm that operates on the Boosting strategy. By integrating multiple weak classifiers, GBDT aims to iteratively improve model performance through a sequential learning process. The algorithm begins by constructing an initial decision tree using the original prediction features. In each subsequent iteration, a new weak classifier is trained to minimize the loss function of the current ensemble model. This is achieved by updating the model in the direction of the negative gradient of the loss function, ensuring a progressive reduction in prediction errors and enhancing overall model accuracy. Through iterative optimization, the residuals progressively converge to zero, and the final prediction is obtained by aggregating the outputs of all individual trees in the ensemble [33]. In this study, the grid search method integrated in scikit-learn library in Python was employed to optimize the parameters of the Gradient Boosting Decision Tree (GBDT) algorithm. The n_estimators parameter was set to 500, the learning_rate to 0.1, the maximum depth to 3, and the random_state to 1.

#### 2.3.3. Fusion model.

**Fig 5:**
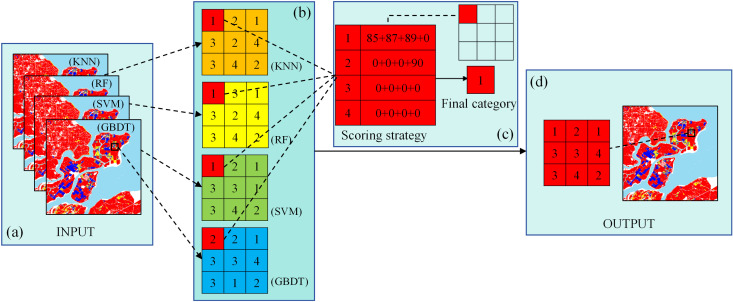
The diagram of the scoring strategy.

This study proposes a novel fusion model based on a scoring strategy that integrates the classification results and validation accuracy of the aforementioned four machine learning algorithms to determine the final category of each pixel (Fig 5). The core idea of the model is to utilize the validation accuracy of each algorithm as a weight, optimizing the classification results through a weighted scoring mechanism to improve classification accuracy and reliability. The specific working principle of the scoring strategy is as follows: first, the classification results of the four machine learning algorithms are input into the scoring mechanism. The Overall Accuracy (OA) of each algorithm on the validation set is used as the weight for its corresponding classification result. For each target pixel, the weights of the same rice types are summed based on the classification results from different algorithms, which yield a comprehensive score for that type. Finally, by comparing the comprehensive scores of different rice types, the type with the highest score is determined as the final rice category for the target pixel. assuming the validation accuracies of the four machine learning algorithms are 85%, 87%, 89%, and 90%, respectively, and the classification results for a certain pixel are Type 1, Type 1, Type 1, and Type 2, the comprehensive score for Type 1 would be 85 +  87 +  89 +  0 =  261, while the score for Type 2 would be 0 +  0 +  0 +  90 =  90. The scores for Type 3 and Type 4 would both be 0. Therefore, the final classification result for this pixel is Type 1, as it has the highest comprehensive score.

### 2.4. Model performance

In the study, the performance of the aforementioned models was evaluated using four key metrics: Overall Accuracy (OA), Kappa coefficient, Mapping Accuracy (MA), and User Accuracy (UA). Overall Accuracy is a commonly adopted metric for assessing model performance, defined as the ratio of correctly classified samples to the total number of samples. The Kappa coefficient serves as a robust measure of classification consistency, providing a comprehensive evaluation of model accuracy. Mapping Accuracy refers to the proportion of correctly predicted samples relative to the total number of actual samples within a specific category. A lower Mapping Accuracy indicates a higher likelihood that other crops types are misclassified into that category. User Accuracy represents the proportion of correctly predicted samples relative to the total number of predicted samples within a specific category. A lower UA suggests a higher probability of samples from that category being misclassified into other categories. The detail formulas are as follows:


$$OA=\frac{\sum_{i=1}^{n}m_{i}}{N}\times 100\%$$ (2)

$$Kappa=\frac{N\cdot \sum_{i=1}^{n}m_{i}-\sum_{i=1}^{n}\text{(}G_{i}\times C_{i}\text{)}}{N^{2}-\sum_{i=1}^{n}\text{(}G_{i}\times C_{i}\text{)}}$$ (3)

$$MappingAccuracy=\frac{m_{i}}{G_{i}}\times \text{100\%}$$ (4)

$$UserAccuracy=\frac{m_{i}}{C_{i}}\times \text{100\%}$$ (5)

where the $m_{i}$ is the number of samples correctly classified. The $G_{i}$ is the number of actual samples of category *i*. The $C_{i}$ is the number of predicted samples of category *i*. The n and N are the total number of categories and the total number of samples, respectively.

### 2.5. Analyzing the driving factors of spatio-temporal changes for rice planting structure

Finally, the classification method based on the scoring strategy was applied to extract the rice planting structure in the Poyang Lake region from 2018 to 2023, followed by the calculation of the rice cultivation area for each county or district. Regression analysis was employed to determine the total area of rice cultivation, the area and proportion of various rice types evolve over time. Additionally, this method was utilized to investigate the correlation between the planting proportion of different rice types and terrain slope, as well as to analyze the annual changes in the average elevation of the distribution of different rice types. Pearson correlation analysis for evaluating the relationships between the planting proportion of different rice types and natural or social factors.

## 3. Results

### 3.1. Comparison of model performance

The Overall Accuracy and Kappa coefficient of different classification methods are shown in Table 2. All four common machine learning algorithms achieved satisfactory classification performance, with 85.29% for KNN, 87.33% for RF, 89.16% for SVM and 90.07% for GBDT, respectively. Notably, the classification method based on scoring strategy further improved the Overall Accuracy to 93.10%, demonstrating its superior ability to integrate the strengths of KNN, RF, SVM, and GBDT, thereby achieving optimal model performance. The Kappa coefficient exhibited a consistent trend with the OA across the evaluated models, ranked in descending order as follows: scoring strategy (0.899), GBDT (0.855), RF (0.843), SVM (0.817), and KNN (0.786).

**Table 2:** Overall accuracy and Kappa coefficient of each classifier algorithm.

Method	Overall Accuracy (%)	Kappa coefficient
K-Nearest Neighbors	85.29	0.786
Random Forest	87.33	0.817
Support Vector Machine	89.16	0.843
Gradient Boosting Decision Tree	90.07	0.855
Scoring Strategy	93.10	0.899

**Fig 6:**
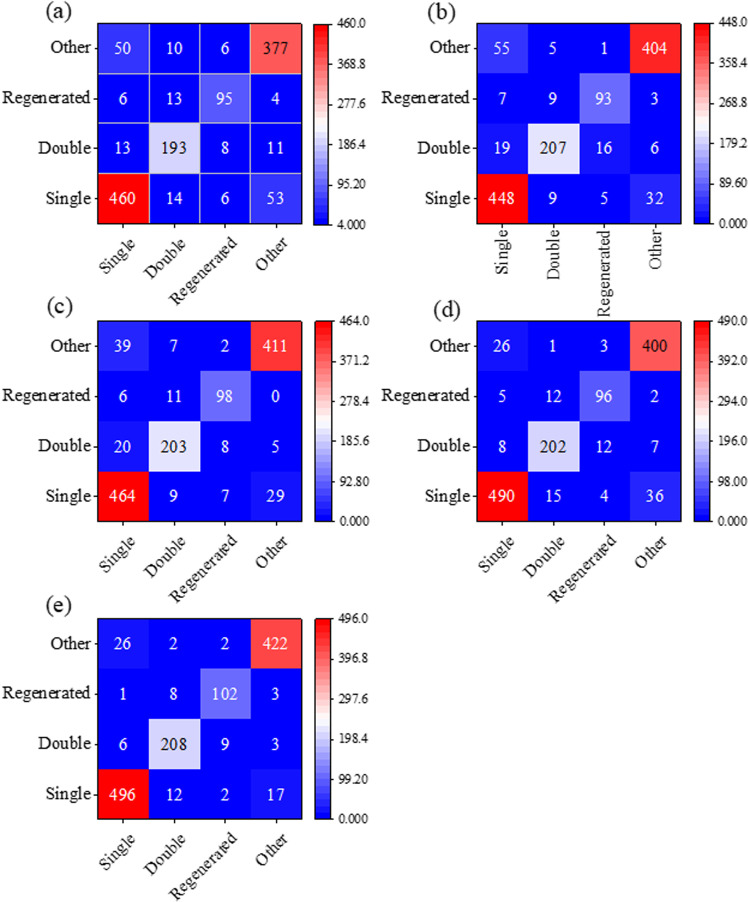
The confusion matrix results of four machine learning and scoring strategy algorithms. (a) K-Nearest Neighbors, (b) Random Forest, (c) Support Vector Machine, (d) Gradient Boosting Decision Tree, (e) Scoring Strategy. Single: single-cropping rice, Double: double-cropping rice, Regenerated: regenerated rice, Other: other land types.

The comparative analysis of classification performance, as depicted in Fig 6, demonstrates that the scoring strategy consistently outperformed individual machine learning models across all rice categories. Furthermore, The comprehensive evaluation, based on confusion matrices, revealed significant improvements in both User Accuracy and Mapping Accuracy when employing the scoring strategy, as detailed in Table 3. Specifically, the User Accuracy metrics for individual machine learning algorithms showed the following ranges across different land types: 86.30%–91.16% for single-cropping rice, 83.47%–88.21% for double-cropping rice, 80.51%–85.22% for regenerated rice, and 85.10%–93.02% for other land types. The scoring strategy achieved superior performance with user accuracies of 94.12%, 92.03%, 89.47%, and 93.36% for these respective categories, representing the highest classification accuracy among all evaluated methods. Similarly, the Mapping Accuracy assessment revealed comparable trends. The four machine learning algorithms exhibited Mapping Accuracy ranges of 84.69%–92.63% for single-cropping rice, 83.91%–90.00% for double-cropping rice, 80.87%–85.22% for regenerated rice, and 84.72%–92.36% for other land types. The scoring strategy again demonstrated superior performance, achieving the highest mapping accuracies of 93.76%, 90.43%, 88.70%, and 93.36% for these respective categories. The analysis identified that classification challenges primarily emerged between crop types with similar NDVI temporal characteristics. Notably, the most frequent classification confusions occurred between single-cropping rice and other crop types, as well as between double-cropping rice and regenerated rice, highlighting the importance of temporal pattern differentiation in crop classification tasks.

**Table 3:** User accuracy and mapping accuracy of each classifier algorithm and scoring strategy (%).

Evaluation type	Classifier type	Single	Double	Regenerated	Other
User Accuracy	K-Nearest Neighbors	86.30	85.78	80.51	85.10
	Random Forest	90.69	83.47	83.04	86.88
	Support Vector Machine	91.16	86.02	85.22	89.54
	Gradient Boosting Decision Tree	89.91	88.21	83.48	93.02
	Scoring Strategy	94.12	92.03	89.47	93.36
Mapping Accuracy	K-Nearest Neighbors	86.96	83.91	82.61	84.72
	Random Forest	84.69	90.00	80.87	90.79
	Support Vector Machine	87.71	88.26	85.22	92.36
	Gradient Boosting Decision Tree	92.63	87.83	83.48	89.89
	Scoring Strategy	93.76	90.43	88.70	93.36

Note: Single: single-cropping rice; Double: double-cropping rice; Regenerated: regenerated rice; Other: other land types.

### 3.2. The spatiotemporal distribution and driving factors of different types of rice

**Fig 7:**
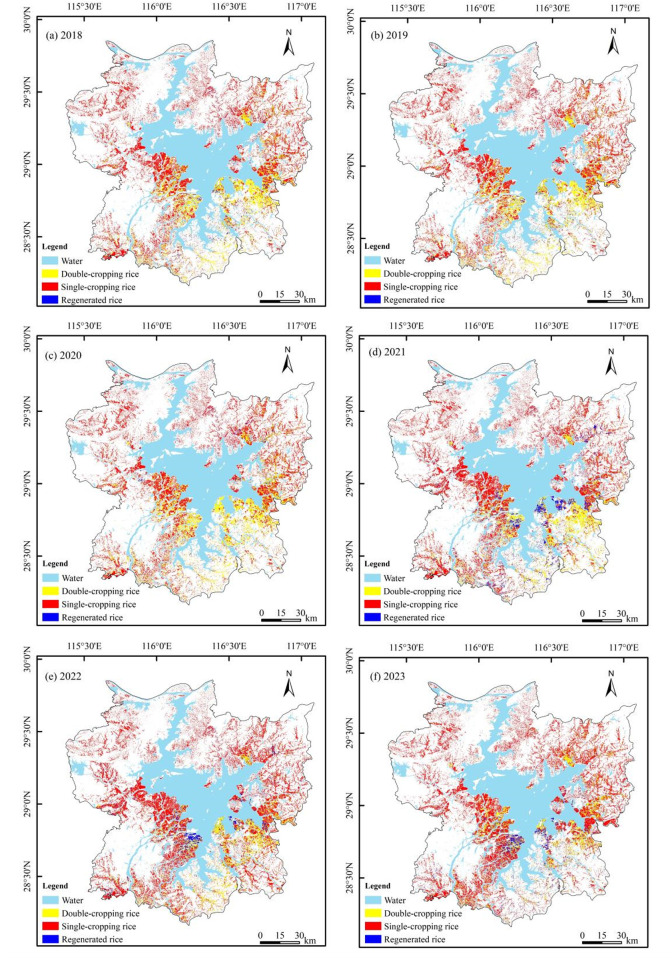
Spatial distribution map of rice planting structure in Poyang Lake area from 2018 to 2023.

**Fig 8:**
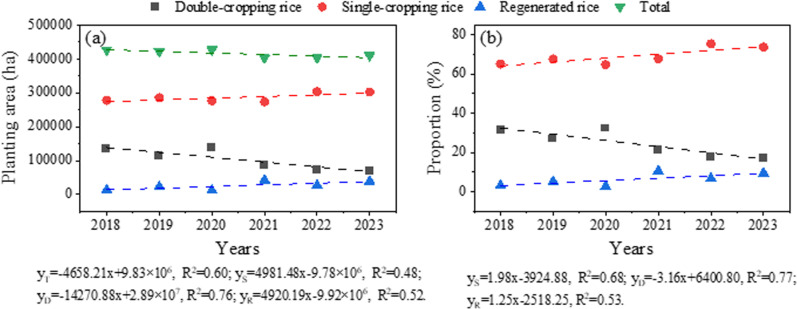
The planting area and total planting area of different types of rice (a), and the proportion of different types of rice planting area (b) from 2018–2023.

The spatial distribution of rice planting structures in the Poyang Lake region from 2018 to 2023 was mapped based on the classification method of scoring strategy (Fig 7), and detailed planting area statistics for single-cropping rice, double-cropping rice and regenerated rice across various counties and districts are shown in Table S1 in S1 File. The analysis revealed distinct geographical patterns in rice cultivation: the northwestern lake region, characterized by mountainous and hilly terrain, was predominantly cultivated with single-cropping rice, while the southeastern plain areas primarily supported double-cropping rice and regenerated rice cultivation. The temporal analysis of rice planting areas over the six-year period (Fig 8) demonstrated significant variations in cultivation patterns. Single-cropping rice maintained the largest cultivation area, ranging from 2.73–3.04 × 10⁵ ha, accounting for 64.78%–75.42% of the total planting area. Double-cropping rice cultivation spanned 0.77–1.39 × 10⁵ ha (17.27%–32.43% of total area), while regenerated rice occupied 1.19–4.25 × 10⁴ ha (2.79%–10.55% of total area). The temporal trends revealed notable patterns: single-cropping rice and regenerated rice showed consistent area expansion, whereas double-cropping rice exhibited a gradual decline throughout the study period. Despite these compositional changes, the total rice cultivation area remained relatively stable at 4.03–4.27 × 10⁵ ha, though a slight decreasing trend emerged from 2021 onward. These spatial and temporal patterns provide valuable insights into the evolving agricultural landscape of the Poyang Lake region.

**Fig 9:**
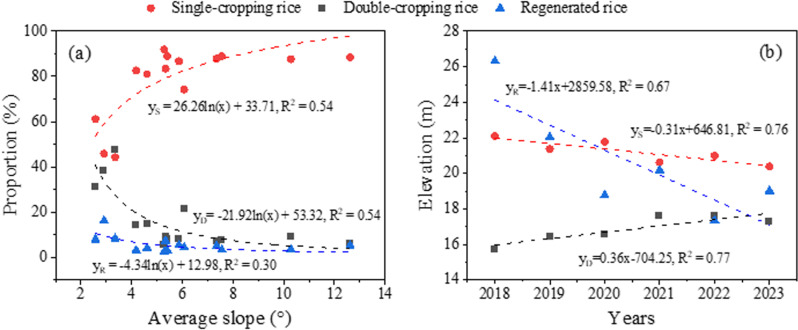
The regression relationship between the average slope in each county or district and the proportion of several rice areas in corresponding regions (a). The trend of changes in the average elevation of planting areas for different rice varieties over time (b).

The spatial distribution and temporal dynamics of rice planting structures are illustrated in Fig 9, revealing distinct patterns and evolutionary trends. The analysis of terrain influence (Fig 9a) demonstrates a strong positive correlation between single-cropping rice cultivation and slope gradient, with its proportion significantly increasing with terrain steepness. Conversely, double-cropping rice and regenerated rice exhibit an inverse relationship, showing decreasing proportions as slope increases. Temporal analysis of elevation patterns (Fig 9b) reveals notable shifts in cultivation zones over time. Single-cropping rice and regenerated rice have shown a gradual downward migration in their average distribution elevation, while double-cropping rice has exhibited an upward elevational shift. These spatial-temporal patterns indicate that the relatively flat terrain surrounding the lake and in the southeastern region has been predominantly utilized for double-cropping rice and regenerated rice cultivation. In contrast, the mountainous and hilly areas farther from the lake have remained strongholds for single-cropping rice. Recent years have witnessed significant spatial redistribution of rice cultivation patterns. Single-cropping rice and regenerated rice have expanded their presence into the flatter lake periphery and southeastern regions, while double-cropping rice cultivation has shown a contracting pattern, increasingly concentrated near the lake areas.

**Fig 10:**
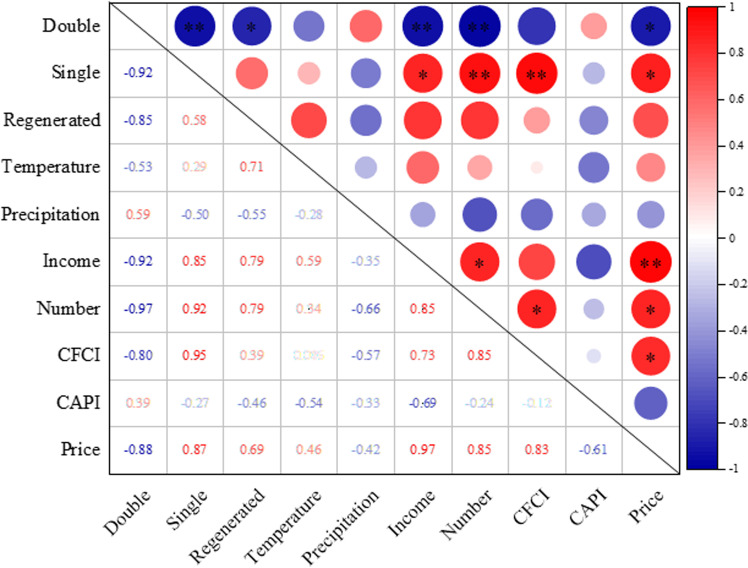
Pearson correlation analysis for the relationships between the proportion of different types of rice planting area and natural factors or social factors. Doble: double-cropping rice; Single: single-cropping rice; Regenerated: regenerated rice; Other: other land types; Income: average income of migrant workers; Number: the total number of migrant workers nationwide; CFCI: China fertilizer wholesale price composite index; CAPI: China agrochemical price index. Price: minimum purchase price of rice. *  *p* <  0.05, ** *p* <  0.01, *** *p* <  0.001.

The correlation analysis revealed distinct patterns in the relationship between rice planting structure and socio-economic factors, while showing no significant association with natural factors (Fig 10). The analysis identified strong correlations with key social indicators, particularly the average income of migrant workers, the total number of migrant workers nationwide, and the Chemical Fertilizer Composite Index (CFCI). Detailed examination of these relationships showed that the proportion of single-cropping rice exhibited significant positive correlations with three key factors: the average income of migrant workers (r =  0.85, p <  0.05), the total number of migrant workers (r =  0.92, p <  0.01), and the minimum purchase price of rice (r =  0.87, p <  0.05). Conversely, double-cropping rice demonstrated significant negative correlations with these same factors. Furthermore, single-cropping rice showed a significant positive correlation with CFCI (r =  0.95, p <  0.01), while double-cropping rice exhibited a negative correlation. Although regenerated rice followed a similar trend to single-cropping rice in response to these social factors, the correlations did not reach statistical significance. These findings suggest that the increasing income levels and growing numbers of migrant workers have created rural labor shortages, driving a shift toward less labor-intensive rice cultivation systems. The expansion of single-cropping rice and regenerated rice areas appears to be a strategic response to these labor constraints, as these systems require less time and labor input compared to double-cropping rice. Additionally, rising fertilizer prices, as reflected in the CFCI, have further incentivized farmers to adopt single-cropping systems, which entail lower fertilizer costs. Notably, despite the implementation of supportive policies such as increased minimum purchase prices for rice, these measures have not been sufficient to reverse the declining trend in double-cropping rice cultivation.

## 4. Discussion

### 4.1. The performance of different classifier models in extracting rice planting structure

The classification of crop planting structures in complex agricultural environments plays a pivotal role in effective agricultural production management. Numerous studies have demonstrated the potential of combining various machine learning algorithms with satellite remote sensing data for crop area extraction, achieving varying degrees of success across different regions [15,16,34,35]. In line with these findings, our study revealed that the integration of commonly used machine learning algorithms (KNN, RF, SVM, GBDT) with Sentinel-2 multi-temporal data resulted in Overall Accuracy ranging from 85.29% to 90.07% and Kappa coefficients between 0.786 and 0.855 for rice planting structure extraction in the Poyang Lake region. To address the limitations of single-algorithm approaches, recent research has explored the integration of multiple machine learning, albeit with increased computational complexity. Several studies have confirmed that ensemble learning methods, which combine multiple machine learning algorithms, can achieve superior accuracy in land cover classification compared to single-algorithm approaches [23,36]. Building upon these advancements, we developed a novel classification method incorporating a scoring strategy derived from multiple individual machine learning algorithms. This innovative approach demonstrated significant improvements over traditional single-algorithm methods, enhancing Overall Accuracy by 3.36%–9.16% and Kappa coefficient by 5.15%–14.38%. Our findings align with the work of Yu et al., who proposed a fusion method based on multiple single-algorithm classification results for winter wheat area extraction in Shandong Province, achieving similarly enhanced classification accuracy [37]. Moreover, our approach distinguishes itself through the implementation of a comprehensive scoring strategy calculated from the validation accuracy of individual machine learning models. This methodology offers greater reliability compared to conventional ensemble learning methods that directly utilize classification results or probabilities from individual models [23,36], providing a more robust framework for crop classification in complex agricultural landscapes. In the future, it will be possible to construct a fusion model based on a complex scoring strategy that integrates deep learning and traditional machine learning with multi-source remote sensing data [4,38,39]. This model will enable precise monitoring of crop growth conditions, identification of crop diseases and pests, prediction of crop yields, and mapping of land cover.

### 4.2. The driving factors of spatiotemporal changes in rice planting structures

The temporal and spatial dynamics of the crop planting structure in a region are profoundly influenced by both natural environmental conditions and socioeconomic factors [7]. In the study, terrain characteristics emerged as a significant determinant affecting the spatial distribution of rice cropping patterns. Specifically, our analysis revealed a positive correlation between terrain slope and the proportion of single-cropping rice cultivation, while the proportions of double-cropping rice and regenerated rice demonstrated an inverse relationship with increasing slope (Fig 9). This spatial pattern can be attributed to several key factors: In hilly and mountainous regions distant from Poyang Lake, the limited accessibility to lake water resources for irrigation has led to the predominance of single-cropping rice, which requires less water input. This finding aligns with Chen et al.‘s research, which identified water resource availability as a crucial driver of cropping pattern modifications [13]. Moreover, the fragmented and non-contiguous nature of paddy fields in these elevated areas increases labor costs and hinders mechanized farming operations, making single-cropping rice a more practical choice for local farmers due to its lower labor and time requirements. On the contrary, the flat topography and abundant water resources in the Poyang Lake periphery and southeastern regions have facilitated the establishment of extensive double-cropping rice cultivation systems. To enhance agricultural productivity in hilly and mountainous areas, strategic interventions are recommended, including the development of water storage infrastructure and the consolidation of small, fragmented rice fields. These measures would not only ensure reliable water supply but also reduce cultivation costs [40], potentially encouraging local farmers to adopt double-cropping systems. Such agricultural intensification could significantly boost regional grain production and contribute to food security objectives.

The study revealed significant shifts in rice cultivation patterns in the Poyang Lake region from 2018 to 2023, characterized by a substantial decrease in both the planting area and proportion of double-cropping rice, accompanied by a marked increase in single-cropping and regenerated rice cultivation. Notably, the spatial distribution patterns showed that single-cropping and regenerated rice cultivation expanded towards the flat areas surrounding the lake and the southeastern region, while double-cropping rice cultivation exhibited a contraction trend towards the lake periphery. These findings are consistent with Huang et al.‘s research, which documented a large-scale conversion from double-cropping to single-cropping rice across Jiangxi Province from 2016 to 2020, resulting in a significant reduction in double-cropping rice area and a corresponding increase in single-cropping rice cultivation [41]. Moreover, this conversion pattern has been widely observed throughout southern China [42]. Specifically, the proportion of single-cropping rice showed positive correlations with migrant workers’ average income, total number of migrant workers, and CFCI, while double-cropping rice proportions exhibited negative correlations with these indicators. The underlying drivers of these changes can be attributed to several interrelated factors: The sustained increase in both the number and income levels of migrant workers in recent years has triggered significant rural labor migration and reduced agricultural labor availability [43]. This phenomenon has created a labor competition between off-farm employment and agricultural production, exacerbating the aging of the agricultural workforce [44], which was the key factor driving the transition from double-cropping to single-cropping and regenerated rice systems [42,45]. Moreover, while grain prices have experienced modest increases, the substantial rise in fertilizer prices has disproportionately increased the production costs of double-cropping rice, making single-cropping rice a more economically viable option for farmers. These shifting patterns pose significant challenges to regional rice production and food security. To address these issues, a comprehensive policy framework is recommended, including: (1) increasing the guaranteed purchase price of rice, (2) implementing targeted subsidies for double-cropping rice cultivation, and (3) providing fertilizer purchase subsidies. Such measures would enhance farmers’ incentives for double-cropping rice cultivation, ultimately boosting rice production and ensuring food security in the Poyang Lake region.

## 5. Conclusions

This study proposed an innovative fusion model based on a scoring strategy that integrates classification results and their validation accuracy from multiple machine learning algorithms and multi-temporal Sentinel-2 data to extract rice planting structure in the Poyang Lake region from 2018 to 2023. Furthermore, we systematically analyzed the key driving factors behind the spatiotemporal dynamics of rice cultivation patterns in this region. The main findings are as follows: (1) The proposed fusion model demonstrated superior performance compared to individual machine learning classification methods. Specifically, it achieved significant improvements across multiple evaluation metrics: Overall Accuracy increased by 3.36%–9.16%, Kappa coefficient by 5.15%–14.38%, User Accuracy by 0.37%–11.13%, and Mapping Accuracy by 0.48%–10.71%. (2) Spatial analysis revealed distinct expansion patterns of different rice cultivation systems. Single-cropping rice and regenerated rice, traditionally concentrated in mountainous and hilly areas, have progressively expanded towards the flat regions surrounding the lake and the southeastern areas. Conversely, double-cropping rice cultivation has exhibited a contraction trend, increasingly concentrated towards the central lake areas. (3) Temporal analysis of the six-year study period showed a clear shift in cultivation patterns, with single-cropping rice and regenerated rice experiencing substantial increases in both planting area and proportion, while double-cropping rice showed a consistent decline. These changes were primarily driven by socioeconomic factors, particularly the increasing income levels and growing number of migrant workers, coupled with rising fertilizer prices. These factors have collectively contributed to rural labor shortages and elevated production costs, prompting farmers to adopt less labor-intensive cultivation systems. The proposed fusion approach offers an effective and relatively simple methodology for classifying crop planting structures, demonstrating enhanced extraction accuracy. Furthermore, the findings provide valuable baseline data to support local governments in formulating more informed agricultural policies aimed at ensuring regional food security. However, the use of single-source Sentinel-2 data and traditional machine learning methods still faces challenges in extracting complex crop planting structures in regions with adverse atmospheric conditions and high cloud cover. Future research should focus on developing integrated approaches that combine multi-source data and leverage advanced deep learning algorithms to further improve the accuracy and robustness of land feature extraction.

## Supporting information

S1 File Contains S1 Table. The planting area of double-cropping, single-cropping and regenerated rice in different counties and districts in Poyang Lake region from 2018 to 2023.
(DOCX)

S2 File Raw data. (ZIP)
