# Supplementary material for: Tracking and analyzing the spatio-temporal changes of rice planting structure in Poyang Lake using multi-model fusion method with sentinel-2 multi temporal data
Source: PLoS One. 2025 Apr 7;20(4):e0320781. doi: 10.1371/journal.pone.0320781 (PMC11975077; doi:10.1371/journal.pone.0320781)
Supplement: S1 File — The planting area of double-cropping, single-cropping and regenerated rice in different counties and districts in Poyang Lake region from 2018 to 2023. (DOCX) [file pone.0320781.s001.docx]

S1 Table. The planting area of double-cropping, single-cropping and regenerated rice in different counties and districts in Poyang Lake region from 2018 to 2023.

| County | Types | 2018 | 2019 | 2020 | 2021 | 2022 | 2023 |
| --- | --- | --- | --- | --- | --- | --- | --- |
| Chaisang | Double | 73.5 | 948.1 | 1126.4 | 655.1 | 166.7 | 460.6 |
|  | Single | 7827.0 | 6605.5 | 6645.1 | 6407.4 | 7756.3 | 7187.8 |
|  | Regenerated | 188.7 | 496.9 | 289.9 | 988.7 | 48.5 | 422.0 |
| De´an | Double | 111.4 | 986.5 | 877.1 | 693.6 | 221.6 | 675.0 |
|  | Single | 8799.0 | 7965.6 | 8482.1 | 7665.1 | 9388.6 | 8273.0 |
|  | Regenerated | 590.9 | 517.8 | 127.5 | 1123.8 | 73.2 | 544.2 |
| Donghu | Double | 23.1 | 57.3 | 82.7 | 19.4 | 13.0 | 37.6 |
|  | Single | 304.1 | 264.7 | 262.8 | 293.4 | 330.2 | 266.4 |
|  | Regenerated | 21.1 | 25.3 | 2.8 | 34.9 | 6.4 | 44.1 |
| Duchang | Double | 2086.2 | 3614.5 | 4209.3 | 1112.7 | 1102.1 | 2024.8 |
|  | Single | 27237.8 | 25456.4 | 24767.0 | 27268.7 | 26737.4 | 26887.7 |
|  | Regenerated | 555.8 | 770.6 | 875.1 | 1503.6 | 778.9 | 997.2 |
| Gongqingcheng | Double | 269.5 | 756.1 | 1175.2 | 817.5 | 922.5 | 966.5 |
|  | Single | 4818.4 | 4359.1 | 4594.4 | 4590.3 | 4450.5 | 5328.1 |
|  | Regenerated | 286.8 | 243.7 | 184.7 | 145.9 | 106.6 | 71.0 |
| Honggutan | Double | 242.2 | 180.4 | 300.2 | 188.9 | 71.0 | 253.0 |
|  | Single | 2740.8 | 2820.0 | 2768.3 | 2649.4 | 3004.7 | 2068.4 |
|  | Regenerated | 129.1 | 112.8 | 45.9 | 270.6 | 244.1 | 786.0 |
| Hukou | Double | 202.6 | 517.1 | 1334.5 | 409.0 | 344.1 | 408.0 |
|  | Single | 9273.7 | 8698.6 | 7878.4 | 8856.4 | 8396.5 | 8947.6 |
|  | Regenerated | 91.8 | 330.1 | 314.6 | 283.8 | 164.2 | 207.1 |
| Jinxian | Double | 14322.9 | 15925.3 | 19037.6 | 11812.4 | 14622.8 | 11598.0 |
|  | Single | 18719.0 | 16222.5 | 15517.2 | 15732.1 | 20429.2 | 38380.0 |
|  | Regenerated | 5176.4 | 6180.4 | 3733.2 | 10489.5 | 2961.9 | 8318.9 |
| Lianxi | Double | 51.8 | 248.6 | 488.4 | 52.5 | 63.0 | 139.3 |
|  | Single | 2062.4 | 1626.7 | 1600.6 | 2032.9 | 2091.9 | 2025.2 |
|  | Regenerated | 105.6 | 336.0 | 124.5 | 22.0 | 66.1 | 55.9 |
| Lushan | Double | 760.0 | 521.5 | 760.1 | 370.2 | 401.2 | 897.6 |
|  | Single | 6177.7 | 6615.8 | 6412.2 | 6409.7 | 6254.1 | 5243.0 |
|  | Regenerated | 162.5 | 144.9 | 104.1 | 495.3 | 401.6 | 145.5 |
| Nanchang | Double | 36269.9 | 26539.7 | 29311.9 | 17953.6 | 12215.4 | 14816.7 |
|  | Single | 42729.3 | 52048.6 | 50870.9 | 38578.5 | 40862.2 | 57062.2 |
|  | Regenerated | 1617.2 | 2033.5 | 445.1 | 8054.9 | 9487.8 | 8755.5 |
| Poyang | Double | 29447.2 | 21320.3 | 25888.0 | 13192.4 | 10857.0 | 9789.3 |
|  | Single | 56414.8 | 59313.5 | 57611.1 | 65021.7 | 70359.6 | 74906.8 |
|  | Regenerated | 1094.0 | 2815.4 | 2944.6 | 6456.0 | 4486.8 | 4941.6 |
| Qingshanhu | Double | 161.9 | 176.7 | 84.7 | 32.7 | 140.9 | 242.5 |
|  | Single | 1271.9 | 1229.9 | 1433.7 | 1211.4 | 1275.7 | 1186.0 |
|  | Regenerated | 85.8 | 110.2 | 4.7 | 275.9 | 104.5 | 90.0 |
| Xinjian | Double | 8067.1 | 7841.3 | 11955.6 | 4715.6 | 3803.2 | 6026.8 |
|  | Single | 37918.3 | 37111.0 | 34813.9 | 40861.2 | 39814.9 | 39795.5 |
|  | Regenerated | 1793.7 | 1788.3 | 983.9 | 1198.5 | 2696.6 | 3010.8 |
| Yongxiu | Double | 1864.0 | 3604.9 | 5198.6 | 771.0 | 1176.7 | 2140.7 |
|  | Single | 29143.3 | 27681.6 | 26348.3 | 27587.1 | 28709.3 | 28190.8 |
|  | Regenerated | 435.2 | 1093.1 | 836.2 | 1335.3 | 1374.6 | 1089.5 |
| Yugan | Double | 39104.7 | 28840.1 | 34753.3 | 32387.4 | 23714.8 | 18304.0 |
|  | Single | 22452.8 | 27578.5 | 26852.0 | 18246.7 | 34259.0 | 36587.7 |
|  | Regenerated | 1257.4 | 5289.2 | 909.1 | 9860.2 | 4260.2 | 9446.6 |
